# Characterisation of an autochthonous mouse ccRCC model of immune checkpoint inhibitor therapy resistance

**DOI:** 10.1038/s41598-025-04917-1

**Published:** 2025-06-05

**Authors:** Asin Peighambari, Hsin Huang, Patrick Metzger, Mojca Adlesic, Kyra Zodel, Silvia Schäfer, Philipp Seidel, Lukas M. Braun, Jan Hülsdünker, Wolfgang Melchinger, Marie Follo, Manching Ku, Stefan Haug, Yong Li, Anna Köttgen, Christoph Schell, Dominik von Elverfeldt, Wilfried Reichardt, Robert Zeiser, Mathias Heikenwalder, Rouven Höfflin, Melanie Börries, Ian J. Frew

**Affiliations:** 1 Department of Internal Medicine I, Hematology, Oncology and Stem Cell Transplantation, Faculty of Medicine, Medical Center - University of Freiburg, Freiburg, Germany; 2 Institute of Medical Bioinformatics and Systems Medicine, Faculty of Medicine, Medical Center - University of Freiburg, Freiburg, Germany; 3 Clinic for Pediatric Hematology & Oncology, Faculty of Medicine, Medical Center - University of Freiburg, Freiburg, Germany; 4 Institute of Genetic Epidemiology, Faculty of Medicine, Medical Center - University of Freiburg, Freiburg, Germany; 5 Institute for Surgical Pathology, Faculty of Medicine, Medical Center - University of Freiburg, Freiburg, Germany; 6 Division of Medical Physics, Department of Diagnostic and Interventional Radiology, Faculty of Medicine, Medical Center - University of Freiburg, Freiburg, Germany; 7 Comprehensive Cancer Center Freiburg (CCCF), Faculty of Medicine, Medical Center - University of Freiburg, Freiburg, Germany; 8 Partner Site Freiburg, German Cancer Consortium (DKTK), Freiburg, Germany; 9 Division of Chronic Inflammation and Cancer, German Cancer Research Center (DKFZ), Heidelberg, Germany; 10 Signalling Research Centre BIOSS, University of Freiburg, Freiburg, Germany

**Keywords:** Renal cell carcinoma, Cancer immunotherapy

## Abstract

Many metastatic clear cell renal cell carcinomas (ccRCC) are resistant to immune checkpoint inhibitor therapies, however the mechanisms underlying sensitivity or resistance remain incompletely characterised. We demonstrate that ccRCCs in the *Vhl/Trp53/Rb1* mutant mouse model are resistant to combined anti-PD-1/anti-CTLA-4 therapy alone and in combination with additional therapeutic agents that reflect current ccRCC clinical trials. However, in some animals in vivo checkpoint therapy allowed isolated splenic T cells to recognise cultured ccRCC cells from the same animal, implicating the tumour microenvironment in suppression of T cell activation. We identified putative immunosuppressive myeloid cell populations with features similar to myeloid cells in the microenvironment of human ccRCC. The expression patterns of immune checkpoint ligands in both the mouse model and in human ccRCC suggests that several checkpoint systems other than PD-1 and CTLA-4 are likely to represent the dominant T cell suppressive forces in ccRCC. Our findings characterise an autochthonous mouse ccRCC model of immune checkpoint inhibitor therapy resistance and pave the way for a systematic functional dissection of the identified potential molecular barriers to effective immune therapy of ccRCC.

## Introduction

Kidney cancers represent approximately 2–3% of all human cancers and ccRCC accounts for roughly 70% of all renal malignancies^1^. The use of immune checkpoint therapies for metastatic ccRCC has revolutionised the standard of care of this disease and improved patient outcomes. There are now four approved immune checkpoint combinations based on combined nivolumab (anti-PD-1) + ipilimumab (anti-CTLA-4) therapy or combinations of different multi-tyrosine kinase inhibitors (axitinib, cabozantinib, lenvatinib) together with anti-PD-1 or anti-PD-L1 therapeutic antibodies (reviewed in^2^). While these therapeutic options have greatly improved response rates and have resulted in impressive long term responses and even cures in some patients, the majority of patients sooner or later develop resistance to the therapies and for these patients the disease remains incurable. For example, a long-term follow up of the CHECKMATE-214 study showed that nivolumab + ipilimumab achieved a superior 5 year survival rate of 43% compared to 31% in the sunitinib therapy group in patients that were initially stratified as having an intermediate to poor risk^3^. The fact that the majority of metastatic ccRCCs show some objective responses to the different types of combination immunotherapies demonstrates that at least some form of anti-tumour immune response is activated in larger numbers of patients. It will be important to better understand the factors that govern whether a patient responds to these therapeutic approaches or not, as well as to identify the factors that act to limit or prevent full curative anti-tumour immunity.

Responses to PD-1 immune checkpoint blockade in most solid tumour types correlate with high mutational burden and neoantigen load. However, in ccRCC the total number of non-synonymous mutations, neoantigens, frame shift mutations and degree of CD8^+^ T cell infiltration do not correlate with response to anti-PD-1 therapy^4,5^. Loss of function mutations of *PBRM1* and loss of chromosome 10q23.31 correlate with good responses to anti-PD-1 therapy but neither have large effect sizes, and these associations have not been observed in all studies^4–9^, indicating that the individual genetics of each patient’s ccRCC tumour does not play a dominant role in determining the likelihood of response to currently-available immune checkpoint therapy. Other cell types in the immune microenvironment of ccRCCs may potentially act to suppress T cell activation and influence checkpoint therapy responses. The blood of human ccRCC patients exhibits increased numbers of monocyte myeloid-derived suppressor cells (M-MDSC) and polymorphonuclear myeloid-derived suppressor cells (PMN-MDSC)^10^. In human ccRCC tumours mass cytometry^11^ and RNA analyses^12^ defined immunosuppressive populations of TAM whose abundance correlated with poor patient outcome. Single-cell RNA-sequencing dissection of human ccRCC tumours revealed that a population of tumour-associated macrophages (TAM) that express *TREM2*, *APOE* and *C1Q* genes is associated with recurrence after surgery^13^ and that a similar population of TAM2-like cells is enriched in late stage and metastatic tumours versus early stage tumours^14^, implicating TAMs in processes associated with aggressive ccRCC disease. TAM populations have also been implicated in determining sensitivity and resistance to immune checkpoint therapy in ccRCC^15,16^ and an interaction network between inflammatory macrophages, exhausted CD8 + T cells and presentation of tumour neoantigens has been shown to be predictive of response to immune checkpoint blockade^17^. Co-culture experiments of TAM isolated from human ccRCC tumours confirmed their immunosuppressive activity towards T cells^12^ and co-culture experiments of a human ccRCC cell line with normal peripheral blood monocytes induced the formation of an M-MDSC-like phenotype that was able to suppress T cell proliferation^18^. These findings support the concept that the accumulation of various myeloid-lineage cells in the microenvironment of human ccRCCs might act to inhibit effective T cell-mediated anti-tumour immune responses.

Mechanistic understanding of the ccRCC tumour microenvironment and the factors that influence responses to checkpoint blockade has been limited by the lack of syngeneic and genetically engineered mouse models of this disease. We recently created an autochthonous mouse model of ccRCC^19^ based on the inducible renal epithelial cell-specific (*Ksp1.3-CreER*^*T2*^) deletion of *Vhl, Trp53* and *Rb1* to model genetic disruption of *VHL* together with genetic alterations in the cell cycle network that arise in human ccRCC tumours^19^. Tumours in this immunocompetent model evolved over the course of 25–61 weeks following gene deletion in adult kidneys and exhibited histological, immunohistochemical, transcriptional, proteomic and mutational similarities to human ccRCC^19,20^. ccRCC in this model also showed differing patterns of sensitivity to everolimus and sunitinib^19^, at least partly mimicking the variable responses seen in human ccRCC patients treated with these agents. The most common classes of single nucleotide variations observed in ccRCCs in the mouse model are the same three most commonly arising mutations in human ccRCC, namely C>A/G>T transversions, C>T/G>A transitions and A>G/T>C transitions. ccRCCs in the mouse model exhibit on average 161 non synonymous variations compared to an average of 51 non synonymous variations in human ccRCC^19^, meaning that the model does not oversimplify the genetics of the human tumours in the sense of likely expression of neoantigens, an important factor when studying the anti-tumour immune response. We previously characterised the tumour immune microenvironment in this model by bioinformatic deconvolution of bulk RNA-sequencing combined with quantitative immunohistochemistry, identifying a strong infiltration by myeloid lineage cells, similar to the tumour immune microenvironment of human ccRCC^20^. We also demonstrated using immunohistochemistry that the density of intratumoural CD4 and CD8 T cells in the mouse model was similar to the density in human ccRCC^20^. We further showed that T cell infiltration and activation were affected by the HIF-1α and HIF-2α status of the mouse tumours and that a similar relationship exists in human ccRCC^20^. Thus, the immune microenvironment of the *Vhl/Trp53/Rb1* mutant mouse ccRCC model exhibits many similarities with human ccRCC. In the current study we investigate responses of the *Vhl/Trp53/Rb1* model to immune checkpoint therapy and compare immunosuppressive molecular features of the model to human ccRCC.

## Results

### *Vhl/Trp53/Rb1* mutant ccRCC tumours are resistant to immune checkpoint inhibition therapy

**Fig. 1:**
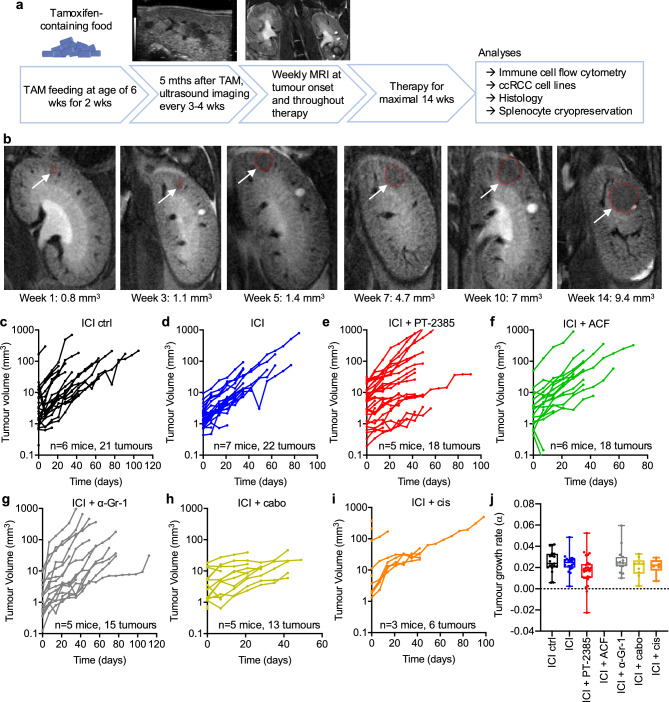
Immune checkpoint therapy for VpR ccRCC tumours. (**a**) Overview of the experimental protocol for generating and monitoring ccRCC tumours before and during therapy. (**b**) Example of an MRI imaging series of an animal treated with anti-PD-1 + anti-CTLA-4 showing tumour development over a period of 14 weeks and associated tumour volumes. Arrows and red circles highlight the tumour in each image. (**c**–**i**) Tumour volume growth curves during treatment with the indicated therapies. ICI: anti-PD-1 + anti-CTLA-4, ICI ctrl: isotype control antibodies, ACF: acriflavine, cabo: cabozantanib, cis: cisplatin. (**j**) Tumour growth rates expressed as the exponential growth co-efficient a where tumour growth is defined by the function e^αt^. Brown-Forsythe and Welch’s ANOVA tests revealed no significant differences in growth rates between any groups.

To model immune checkpoint inhibition therapy of human ccRCC we used an autochthonous mouse model of ccRCC based on tamoxifen-induced gene deletion in *Ksp1.3-CreER*^*T2*^*; Vhl*^*fl/fl*^*; Trp53*^*fl/fl*^*, Rb1*^*fl/fl*^ mice^19^ (hereafter abbreviated as VpR) to test the effects of treatment with isotype control antibodies (hereafter ICI ctrl) or combined anti-PD-1 plus anti-CTLA-4 antibodies (immune checkpoint inhibition, hereafter ICI) (Fig. 1a). Mice were imaged using ultrasound on a monthly basis beginning 5 months after feeding with tamoxifen-containing food to detect tumour onset in each individual animal. Subsequently, magnetic resonance imaging (MRI) was conducted on a weekly basis to monitor tumour progression during therapy. Figure 1b shows an example of tumour development over a 14 week period. We used a combination of anti-PD-1 and anti-CTLA-4 antibody clones and dosing schedule that has previously been shown to be therapeutically efficacious in combination with other agents in a mouse model of prostate cancer^21^. Visual inspection of tumour growth curves (Fig. 1c,d) revealed that ICI therapy did not induce tumour regression nor a slowing in growth. We previously demonstrated that untreated VpR tumours exhibit an exponential growth rate^20^ using the exponential linear regression formula e^αt^ where α describes the coefficient of growth, and t represents time in days after therapy induction, allowing quantification of exponential growth (α) for each tumour (Fig. 1j). This quantitative analysis revealed that ICI therapy was therapeutically ineffective in this model. In a subsequent round of treatment experiments we therefore investigated whether addition of other therapeutic agents might induce a response to ICI therapy. We treated cohorts of mice with ICI in combination with either:

(i) the HIF-2α specific inhibitor PT-2385 (ICI + PT-2385) (Fig. 1e), analogous to the NCT05239728 ccRCC clinical trial testing the combination of pembrolizumab (anti-PD-L1) and belzutifan (HIF-2α inhibitor),
(ii) the dual HIF-1α / HIF-2α inhibitor acriflavine (ICI + ACF) (Fig. 1f),
(iii) anti-Gr-1 antibodies (Fig. 1g) in an unsuccessful attempt to deplete Ly-6C- and Ly-6G-expressing myeloid cells in the tumours (see below),
(iv) the multi-tyrosine kinase inhibitor cabozantinib (ICI + cabo) (Fig. 1h), reflecting the therapeutic combination of the COSMIC-313 ccRCC trial (NCT03937219) or,
(v) the chemotherapeutic drug cisplatin (ICI + cis) (Fig. 1i) to test the effects of potentially immunogenic chemotherapy.

The combination of ICI + cabo was associated with toxic side effects, with 3 of 5 mice having to be sacrificed early due to weight loss, potentially consistent with the higher frequency of grade 3 and 4 adverse effects caused by this triple therapy combination in the COSMIC-313 trial^22^. None of the tested combination therapies altered tumour growth rates (Fig. 1j). The absence of therapeutic responses suggested that the VpR ccRCC model might represent a good system to study the mechanisms underlying intrinsic or acquired anti-PD-1 plus anti-CTLA-4 checkpoint therapy resistance that is a frequent feature of human ccRCC.

### Immune checkpoint therapy does not promote an anti-tumour immune microenvironment

**Fig. 2:**
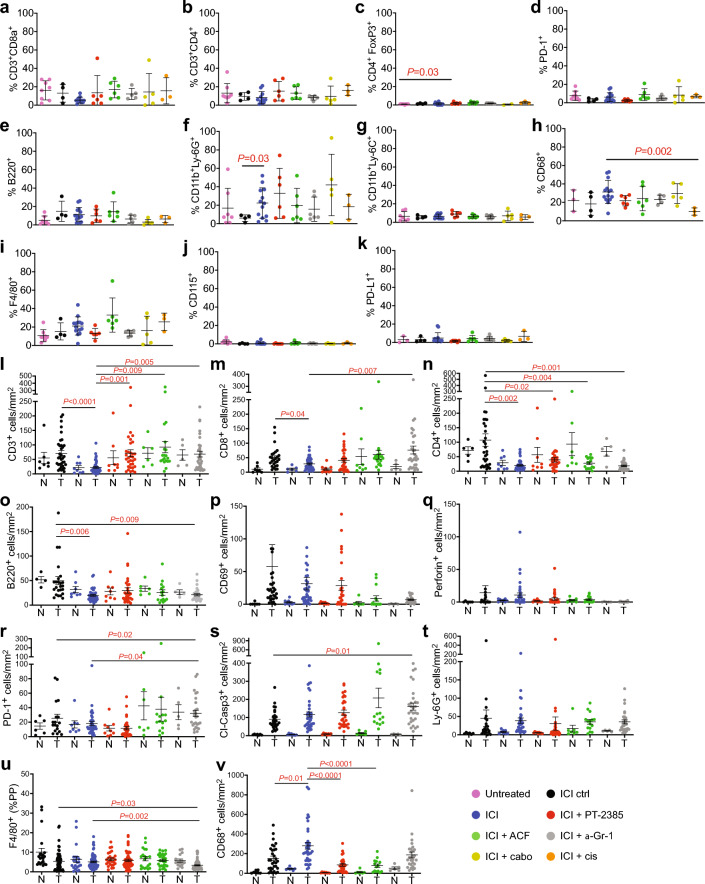
Characterisation of the effects of therapies on the tumour microenvironment. (**a**–**k**) Flow cytometry analyses of cell positive for the indicated markers in the tumour immune microenvironment expressed as percentage of tumour infiltrating CD45^+^ cells in each of the indicated therapy groups. Statistically significant differences between groups were determined using Welch’s ANOVA test followed by Dunnett’s T3 multiple comparisons test. (**l**–**v**) Immunohistochemical analyses of the tumour immune microenvironment. Densities of cells/mm^2^ that express the indicated markers in each of the therapy groups are shown. F4/80 positivity was quantified using percentage positive pixels (%PP). N: adjacent normal tissue, T: ccRCC tumour tissue. Statistical differences between groups were determined using Welch’s ANOVA test followed by Dunnett’s T3 multiple comparisons test.

We next investigated whether the therapies might have altered the immune microenvironment without leading to a therapeutic response. Flow cytometry revealed that ICI therapy or ICI in combination with other agents did not alter the frequencies of intratumoural CD8^+^ effector T cells (Fig. 2a), CD4^+^ helper T cells (Fig. 2b), FoxP3^+^ regulatory T cells (Fig. 2c), PD-1^+^ cells (Fig. 2d), B220^+^ B cells (Fig. 2e), CD11b^+^Ly-6G^+^ granulocytes/putative PMN-MDSCs (Fig. 2f), CD11b^+^Ly-6C^+^ monocytes/putative M-MDSCs (Fig. 2g), CD68^+^ monocytes or macrophages (Fig. 2h), F4/80^+^ differentiated macrophages (Fig. 2i) or CD115^+^ monocytes (Fig. 2j). The single exception was a small reduction of CD68^+^ cells in ICI + cis treated tumours (Fig. 2h). The frequency of cells expressing the checkpoint ligand PD-L1 were very low under all therapeutic combinations (Fig. 2k), consistent with this tumour model not exhibiting a strong PD-L1/PD-1 mediated suppression of T cell activation. Finally, ICI + anti-Gr-1 treatment did not decrease the frequencies of CD11b^+^Ly-6G^+^ or CD11b^+^Ly-6C^+^ expressing cells in tumours (Fig. 2f,g), non-tumoural kidney, spleen, bone marrow and blood (data not shown), revealing that the attempted depletion of these myeloid cells was not successful, likely due to the rapid adaptation of myelopoiesis and the possible formation of neutralising antibodies against the foreign immunodepleting antibody in this immune competent model after long term and repeated administration of the anti-Gr-1 antibody.

While not reaching statistical significance in the sample sizes available, we noted that the ICI treated mice showed a tendency to exhibit a decreased frequency of CD8 + T cells in the tumour immune microenvironment. Since the flow cytometry analyses were limited by the number of tumours in each cohort that had a size that was sufficient to allow isolation of intratumoural immune cells, we next used immunohistochemistry of tumour-bearing kidneys to study the immune microenvironment in a larger number of tumours of different sizes from several different therapeutic cohorts. Adjacent normal tissue was also analysed. ICI reduced the density of intratumoural CD3^+^ T cells (Fig. 2l), CD8^+^ effector T cells (Fig. 2m), CD4^+^ helper T cells (Fig. 2n) and B220^+^ B cells (Fig. 2o) compared to control antibody treatment. Interestingly, the co-administration of either of the HIF-α inhibitors PT-2385 or ACF blocked the effects of ICI treatment on the abundance of these cell types. ICI therapy did not affect the density of CD69^+^ activated lymphocytes (Fig. 2p), Perforin^+^ activated CD8^+^ T cells (Fig. 2q), PD-1^+^ cells (Fig. 2r) or number of cells staining positively for an antibody specific to cleaved-Caspase 3 to assess the extent of potential immune-mediated apoptosis of tumour cells (Fig. 2s). The densities of Ly-6G^+^ granulocytes (Fig. 2t) and F4/80^+^ differentiated macrophages (Fig. 2u) were unaffected by ICI. In contrast, ICI increased the density of intratumoural CD68^+^ monocytes/macrophages and this increase was also blocked by co-administration of PT-2385 or ACF (Fig. 2v).

Collectively, these findings demonstrate, contrary to the goal of the intervention, that ICI therapy reduces the number of T cells infiltrating ccRCC tumours and highlight that in comparison to normal kidney tissue that ccRCCs are highly infiltrated by different types of myeloid lineage cells, with ICI inducing a moderate increase in CD68^+^ myeloid lineage cells.

### Systemic immune checkpoint therapy enhances T cell recognition of ccRCC cells

**Fig. 3:**
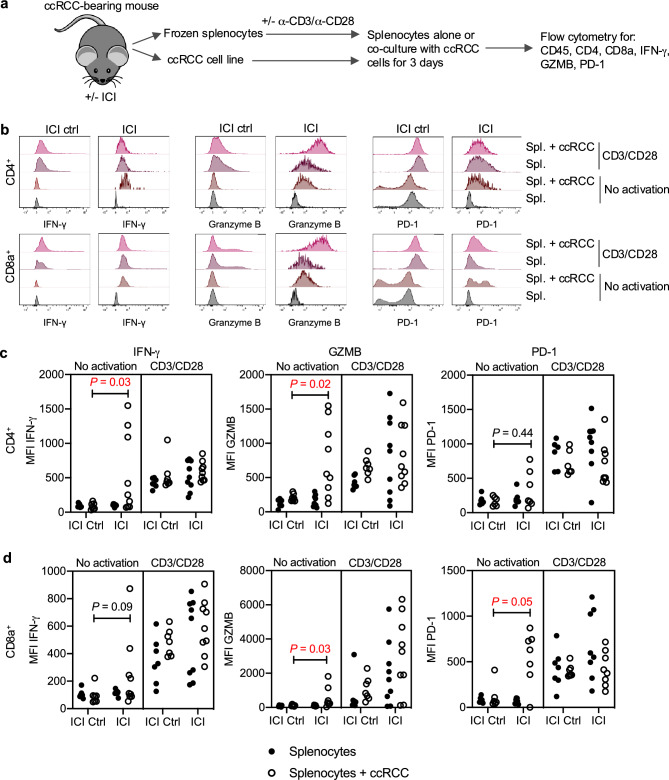
Evidence for recognition of ccRCC tumour cells by peripheral T cells induced by immune checkpoint therapy. (**a**) Overview of the experimental workflow. (**b**) Representative flow cytometry histograms of CD4^+^ and CD8^+^ T cells from an ICI ctrl mouse or an ICI treated mouse, with or without activation by α-CD3/α-CD28 beads and with or without co-culture with ccRCC cells from the same mouse. (**c**,**d**) Summary of the results of the assay for all splenocyte-ccRCC cell line pairs for CD4 ^+^ (**c**) and CD8a ^+^ (**d**) T cells. Each dot represents T cells from an individual animal and shows the median fluorescence intensity (MFI) of the indicated antibody staining. Statistical differences between groups were determined using the non-parametric Mann–Whitney test.

From some treated animals we were able to generate ccRCC cell lines and frozen splenocyte suspensions, representing a small biobank of matched samples. We conducted ex vivo syngeneic T cell/tumour cell mixing experiments designed to determine whether T cells from the spleen have the ability to recognise the tumour cells and whether this is altered by ICI therapy. We reasoned that removing the tumour cells and T cells from the context of a putative myeloid cell-mediated immunosuppressive microenvironment might reveal systemic T cell activation that was not observed in the tumour microenvironment. Since these experiments were limited by the number of mice that had a) sufficiently large tumours to allow sampling for the generation of cell lines and the above-described flow cytometry and immunohistochemical assays and b) those tumours that successfully generated cell lines, we analysed the samples in two groups: 1) Control: mice that were untreated (n = 3) plus mice that were treated with control antibodies (n = 3) and 2) ICI treated: mice that were treated with ICI only (n = 1) or with ICI plus ACF (n = 1), cabo (n = 1), cis (n = 2) or anti-Gr-1 (n = 4). Thawed splenocytes were either not activated or activated with α-CD3/α-CD28 beads as a positive control to establish that the frozen T cells were viable, and cultured alone or co-cultured with the ccRCC cell line derived from the same animal (Fig. 3a). After 3 days, CD4^+^ and CD8^+^ T cells were analysed by flow cytometry to determine their activation state by staining for IFN-γ, GZMB and PD-1. Representative examples of the flow cytometry histograms (Fig. 3b) illustrate that in some of the ICI-treated animals but not in any of the control animals, CD4^+^ and CD8^+^ T cells displayed activation upon exposure to ccRCC cell lines to an extent that is similar to activation observed with α-CD3/α-CD28 beads in the absence of co-culture. CD4^+^ T cells from ICI treated animals showed an increase in IFN-γ and GZMB expression and a non-significant trend towards increased PD-1 expression when co-cultured with ccRCC cells (Fig. 3c). CD8^+^ T cells from ICI treated animals showed an increase in GZMB and PD-1 expression and a non-significant trend towards increased IFN-γ expression when co-cultured with ccRCC cells (Fig. 3d). While the technical limitations of the experiment which led to the pooling of mice with different combination ICI treatments into the same cohort prevent definitive conclusions, these findings provide evidence that anti-PD-1 plus anti-CTLA-4 therapy can enhance T-cell recognition of tumour cells, at least in some animals, evidenced by the increased presence of tumour-reactive T cells in the spleen. We therefore hypothesised that the absence of increased intratumoural T cell numbers or activation in ICI-treated animals suggests that the microenvironment of the ccRCC tumours suppresses local T cell activation and effective anti-tumour immunity.

### Single cell dissection of the mouse ccRCC tumour microenvironment

**Fig. 4:**
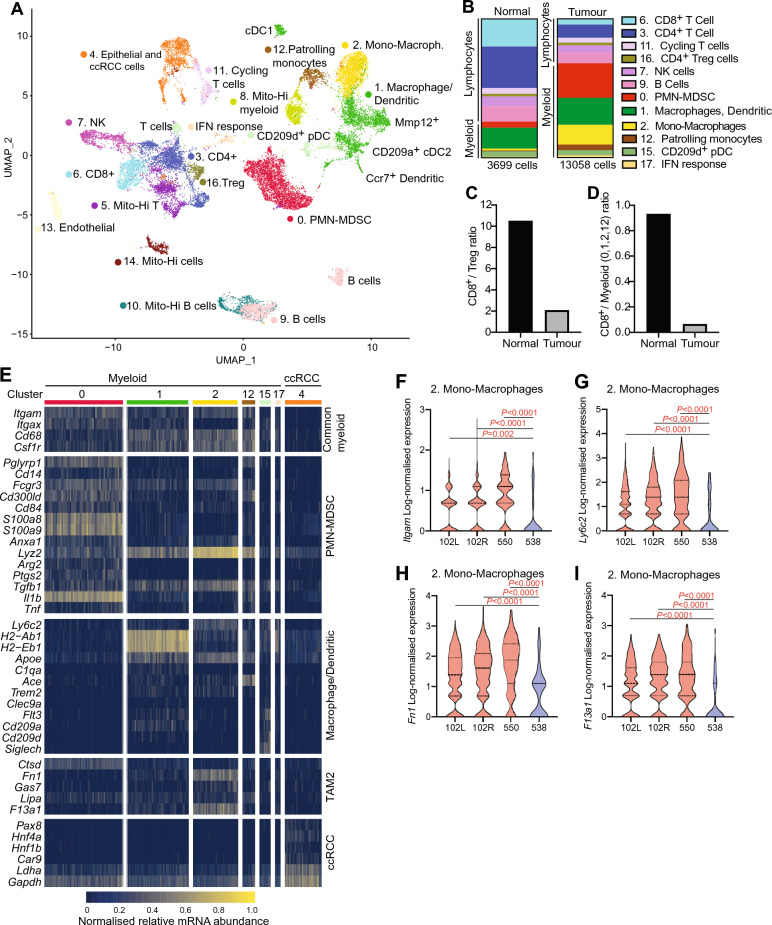
Single cell RNA-seq dissection of the immune microenvironment of VpR ccRCC tumours. (**a**) UMAP plot with identified cellular clusters. (**b**) Abundance of different immune cell types in normal and tumour samples. (**c**) Ratio of CD8^+^ T cells to regulatory T cells in normal and tumour samples. (**d**) Ratio of myeloid cells to CD8^+^ T cells in normal and tumour samples. (**e**) Single cell heatmap of gene expression in the indicated clusters of myeloid lineages and ccRCC cells pooled from three mouse ccRCCs. Each column represents a single cell. (**f**–**i**) Violin plots of abundance of the indicated mRNAs in cells of the indicated clusters. *P* values were calculated using Kruskal-Wallace non-parametric one-way ANOVA with Dunn’s multiple comparisons test.

We next defined the cellular composition and molecular features of tumour cells and immune cells in the tumour microenvironment through single cell RNA-sequencing (scRNA-seq) analyses of three mouse ccRCC tumours derived from two untreated tumour-bearing animals. A renal cortex sample from an age-matched, Cre-negative animal served as a reference allowing assignment of cell cluster identities. Since flow cytometry revealed that immune cells typically represent < 10% of cells in cellular digests of mouse ccRCC tumours we used flow cytometric cell sorting to enrich immune cells from normal and ccRCC samples in the final sequenced population. After digestion of tissue samples, CD45.1^+^ immune cells were mixed in the ratio of 3:1 with CD45.1^-^ cells for preparation of single cell libraries and sequencing. A total of 5151 cells with a median of 1713 identified genes per cell were analysed in the normal kidney sample and 5347 (median 1431 genes per cell), 5300 (median 1280 genes per cell) and 7823 (median 1127 genes per cell) cells were analysed in the three tumour samples, respectively. scRNA-seq datasets were pre-processed with scTransform, integrated and batch corrected using harmony. UMAP projection identified 18 clusters (clusters 0–17) (Fig. 4a and Supplementary Fig. 1a). We combined cluster-specific differential gene expression analyses with cellular lineage markers that were identified in previous scRNA-seq studies of resident renal cells^23,24^, our previous bulk RNA-seq studies of the mouse ccRCC model^19,20^, and well-established marker genes to annotate clusters (Fig. 4a, Supplementary Fig. 1b, Supplementary Fig. 2). Four clusters (5, 8, 10, 14) were excluded from further analyses based on high mitochondrial gene content, likely reflecting dying cells or cells that were damaged during the steps of processing for scRNA-seq. Cluster identities were confirmed with multiple marker genes for each cluster.

ccRCC cells clustered together with normal renal epithelial cells in cluster 4 and expressed proximal tubule markers such as *Hnf1b, Hnf4a, Pax8, Gpx3, Krt8, Slc34a1*. This likely reflects the strong overalp in transcriptional programmes between mouse and human ccRCC cells and their normal proximal tubule counterparts^19^. Nonetheless, differential gene expression analyses clearly segregated the normal and ccRCC populations (Supplementary Fig. 1c). Several HIF-1α target genes (Supplementary Fig. 1d-i) that we had previously identified in the mouse ccRCC model^19,20^ were upregulated in ccRCC cells, confirming that the tumour cells display the characteristic HIF-α-mediated gene expression signature of mouse and human ccRCC.

We next compared the relative frequency of each separate immune cell cluster within the overall immune cell compartment, defined as all *Ptprc* (encoding CD45)-expressing clusters (Fig. 4b), between normal and tumour samples. The tumour immune microenvironment displays a shift in the proportion of lymphocyte and myeloid cells compared to the normal kidney, with a strong relative enrichment of the proportion of myeloid cells from cluster 0 (PMN-MDSC), cluster 1 (macrophages and dendritic cells), cluster 2 (Monocyte-derived macrophages) and cluster 12 (patrolling macrophages). The relative proportions of the different cell types are also suggestive of an immunosuppressive tumour microenvironment. While normal kidney displays a ratio of approximately one Treg cell for every ten CD8^+^ cells, ccRCC tumours have a ratio of one Treg cells for only two CD8^+^ cells (Fig. 4c). Given that myeloid lineage cells infiltrating tumours frequently display immunosuppressive properties, it is also noteworthy that in normal kidney there is a ratio of approximately 1.1 myeloid lineage cell for each CD8^+^ cell, in the ccRCC tumours there are approximately 15 myeloid cells for every CD8^+^ T cell (Fig. 4d).

### T cell activation in mouse ccRCC

We next focused our analyses on specific markers of T cell function. The cycling T cell population (cluster 11) displayed a strong signature of proliferation-associated genes including *Mki67* and *Top2a* and included CD8^+^ and CD4^+^ cells and represented about 18% of the entire T cell population in tumours. Notably, in the normal kidney, the cycling T cell population represented only 7% of the total T cell population, suggesting that there is some degree of T cell proliferation in the tumour microenvironment, potentially consistent with an ongoing anti-tumour T cell-mediated immune response. However, it is also notable that a series of genes that are expressed in activated CD8^+^ T cells (*Gzmb*, *Prf1*, *Il2*, *Ifng*, *Tnf*, *Fasl*) or in activated CD4^+^ T cells (*Tgfb1*, *Cd40lg*, *Il2*, *Ifng*, *Tnf*, *Fasl*) were not upregulated in the relevant CD8^+^ and CD4^+^ clusters in tumours versus normal (Supplementary Fig. 3a,b). There was a very slight increase in expression of some T cell exhaustion genes (*Pdcd1*, *Lag3*, *Eomes*, *Tox*) in the CD8^+^ clusters from tumours versus normal but this was not observed in the CD4^+^ clusters (Supplementary Fig. 3C,D). In summary, while the total numbers of T cells in the ccRCC microenvironment are relatively small, the gene expression patterns are consistent with only a very limited degree of activation of anti-tumour activity and a very weak expression profile of T cell exhaustion.

### Putative immunosuppressive myeloid lineage cells in mouse ccRCC

Analyses of the myeloid lineage cell clusters revealed expression of many different genes that are known to regulate different aspects of suppression of T cell-mediated immunity in ccRCC and other tumour types. The most notable difference between tumour and normal kidney myeloid cell populations is the greatly increased proportional abundance of neutrophil/PMN-MDSC-like cells (cluster 0) and a population of cells expressing several markers of monocytes and macrophages (cluster 2) in tumours (Fig. 3b). Cluster 0 cells exhibited a strong gene expression signature of PMN-MDSCs (including *Pglyrp1*, *Cd14*, *S100a8*, *S100a9*, *Anxa1*, *Mmp8*, *Ltb4r1*)^25^ and high expression of *Cd300ld*, which was recently shown to mediate immune suppression in tumours^26^ (Fig. 4e). Single cell heatmap analyses (Fig. 4e) and UMAP-based gene expression analyses (Supplementary Fig. 4) revealed that cluster 2 cells in tumours expressed common markers of monocyte/macrophage lineage cells (*Cd68, Csf1r, Itgam, Apoe*), as well as the differentiated macrophage marker *Adgre1* (encoding F4/80), but lacked expression of markers of antigen-presenting macrophages that are found in cells in cluster 1 (*Itgax* and MHC class II genes such as *H2-Eb1*)*.* Cluster 2 cells in tumours also expressed higher levels of *Itgam* (Fig. 4f) and *Ly6c2* (Fig. 4g) than cells from normal kidney. These genes encode CD11b and Ly-6C, respectively, and high cell surface expression of these proteins are flow cytometry markers of mouse monocyte-derived macrophages of M-MDSCs, suggesting that cluster 2 in ccRCC tumours may contain a population of immunosuppressive monocyte-derived macrophage-like cells, hereafter termed Mono-macrophages. scRNA-seq dissection of human ccRCC tumours previously revealed that a population of tumour-associated macrophages (TAM) that express *TREM2*, *APOE* and *C1Q* genes is associated with recurrence after surgery^13^ and that a similar population of TAM2-like cells is enriched in late stage and metastatic tumours versus early stage tumours^14^, implicating TAMs in processes associated with aggressive ccRCC disease. Cells expressing these markers in mouse ccRCC were present in the larger macrophage cluster 1 and cells expressing *Trem2* and *Apoe*, but lacking *C1q* genes, were present in the monocyte/macrophage cluster 2 and the patrolling monocyte cluster 12. Genes that were identified as markers of TAM2 in human ccRCC cells, including *Fn1* (Fig. 4e,h)*, F13a1* (Fig. 4e,i) and *Gas7* (Fig. 4e) were more highly expressed in Mono-macrophage cluster 2 cells from tumours than from normal kidneys. In conclusion, the expanded population of Mono-macrophage cells in tumours is consistent with an aberrant state of myeloid cell differentiation that exhibits cellular markers of putative M-MDSCs as well as more differentiated macrophages that express a series of markers that reflect at least some aspects of the TAM cell composition of aggressive human ccRCC tumours. These observations are potentially consistent with this population representing a continuum of aberrant differentiation states along the lineage of patrolling monocytes to M-MDSCs to TAM. Different myeloid cell clusters also variably express a series of additional genes that have been implicated in suppression of T cell activation (Fig. 4e), including *Tgfb1* (TGF-β), *Il1b* (IL-1β), *Ptgs2* (COX-2) and *Tnf* (TNF-α) each in one or more clusters. Expression and secretion of these enzymes and cytokines might potentially play a role in immunosuppression by myeloid cells in mouse ccRCC.

### Immune checkpoint ligands other than those activating PD-1 and CTLA-4 are abundantly expressed in mouse and human ccRCC

**Fig. 5:**
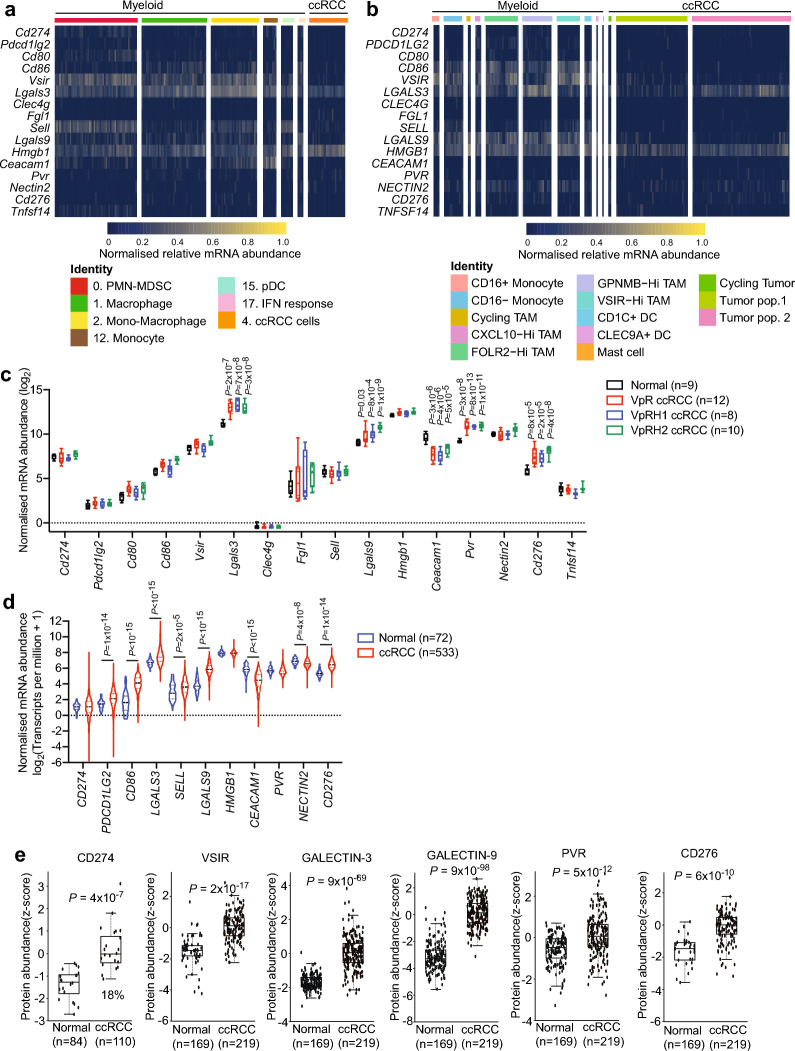
Upregulation of checkpoint ligands in mouse and human ccRCC. (**a**) Single cell heatmap of gene expression in the indicated clusters of myeloid lineages and ccRCC cells pooled from three mouse ccRCCs. Each column represents a single cell. (**b**) Reanalysis of data from^15^ showing single cell heatmap of expression of genes encoding checkpoint ligands in clusters of myeloid lineages and ccRCC cells pooled from eight human ccRCCs. (**c**) mRNA abundance of immune checkpoint ligands in bulk RNA-seq of normal kidney or ccRCCs from *Vhl/Trp53/Rb1* mutant (VpR), *Vhl/Trp53/Rb1/Hif1a* mutant (VpRH1) and *Vhl/Trp53/Rb1/Hif2a* mutant (VpRH2) mice. Boxes show mean and quartiles, whiskers show minimum and maximum values. Statistical differences between tumours of each genotype compared to normal kidney were assessed using unpaired Student’s t-test. (**d**) mRNA abundance of immune checkpoint ligands in bulk RNA-seq of normal kidney or ccRCCs from the TCGA KIRC study. Median and quartile values are shown in the violin plots. Welch’s t-test was used to determine statistical significance. (**e**) Protein abundance of immune checkpoint ligands from the CPTAC ccRCC proteomics study. Welch’s t-test was used to determine statistical significance.

Investigation of the expression of immune checkpoint ligands (Fig. 5a) in the myeloid cell clusters, as well as in the ccRCC cell cluster, revealed that *Cd274* and *Pdcd1lg2* (encoding PD-L1 and PD-L2, respectively) were very weakly expressed. *Cd80* and *Cd86* encode ligands for CTLA-4. *Cd80* was expressed by cells in the PMN-MDSC cluster, while *Cd86* is expressed by cells in clusters the monocyte and macrophage clusters 1, 2, 12 and 17. Genes encoding other checkpoint ligands including *Vsir* (VISTA), *Lgals3* (GALECTIN-3), *Sell* (L-selectin), *Lgals9* (GALECTIN-9), *Hmgb1* (High mobility group box 1) and *Ceacam1* (CEA cell adhesion molecule 1) were highly expressed in different clusters of myeloid cells. *Lgals3* and *Hmgb1* expression are also highly expressed in the tumour cell population. We also observed a very similar pattern of expression of immune checkpoint ligands by reanalysing a previously published scRNA-seq study of 8 human ccRCC tumours^15^ (Fig. 5b). *CD274* and *PDC1LG2* expression levels were very low in myeloid cells and ccRCC cells, *CD86*, *VSIR* and *LGALS9* were high in myeloid cells and *LGALS3* and *HMGB1* were high in both myeloid and ccRCC cells. Analyses of bulk RNA-seq of normal mouse kidney and ccRCCs from *Vhl/Trp53/Rb1*, *Vhl/Trp53/Rb1/Hif1a* and *Vhl/Trp53/Rb1/Hif2a* mice^20^ revealed that the *Lgals3*, *Lgals9*, *Pvr* and *Cd276* checkpoint ligands were upregulated in ccRCCs versus normal tissue and the elevated expression was not altered by the absence of HIF-1α or HIF-2α (Fig. 5c). Analyses of bulk RNA-seq of human ccRCC revealed that *PDCD1LG2* and *CD80* expression were upregulated in ccRCC tumours compared to normal kidney, as was expression of *LGALS3*, *LGALS9*, *SELL* and *CD276* (Fig. 5d). Notably, the expression levels of these latter four genes encoding non-PD-1/CTLA-4 checkpoint ligands were substantially higher than abundance of the PD-1 ligand *PDCD1LG2* and the CTLA-4 ligand *CD80*. To corroborate these data at the protein level we interrogated the CPTAC database of proteomic analyses of normal and human ccRCC samples^27^. While CD274 (PD-L1) expression was detected in only 18% of tumour samples, and PD-L2, CD80 and CD86 were not detected in any samples, upregulation of VSIR, GAL-3, SELL, GAL-9, PVR and CD276 was observed in ccRCCs (Fig. 5e). In summary, these data imply that the different myeloid lineage cells and tumour cells in the ccRCC tumour microenvironment in the mouse model and in humans could potentially contribute to suppression of T cell activation via a series of checkpoint ligand-checkpoint receptor interactions. Ligands for LAG-3, TIM-3 and TIGIT are more strongly and widely expressed than those for PD-1 and CTLA-4, consistent with the observed resistance to anti-PD-1 plus anti-CTLA-4 therapy in the mouse model.

## Discussion

Despite the paradigm-changing nature of immune checkpoint-based therapies for metastatic ccRCC, and the improved 5-year survival rates and even longer-term survival for some patients, for the majority of patients these therapies fail to cure the disease^28^. It remains unclear why some ccRCCs respond well and others respond poorly to checkpoint therapies targeting PD-1 and/or CTLA-4. Studies of the genetic features and analyses of the tumour immune microenvironments of human ccRCCs have revealed that while there are some factors that correlate with clinical responses to immune checkpoint blockade, these are relatively weak in terms of overall effect size^4,5,15,16^. Remarkably, the initial clinical trials, and now the routine clinical application, of PD-1 and CTLA-4 immune checkpoint therapies for ccRCC treatment were never based on clear pre-clinical evidence that was directly related to this tumour entity, due to the absence of appropriate mouse models. The same can be said of the many recently completed and ongoing clinical trials for ccRCC (reviewed in^28^), which aim to target different aspects of the immune microenvironment based on hypotheses that arise from the study of human ccRCC, as well as by inference from studies in other tumour types. This problem of lack of ccRCC-specific pre-clinical evidence for the likely efficacy of ccRCC immunotherapies has been entirely due to the absence of appropriate immunocompetent mouse models that accurately reproduce the salient genetic and immunological features of the human disease. In comparison to other major human tumour types for which genetically engineered, immunocompetent mouse models have been available for decades, the first mouse ccRCC models were developed only in the last 6–8 years^29–33^ and until very recently, none had been used to test immune checkpoint therapies. Unlike most other tumour types, there are also no accurate syngeneic transplantable mouse models of ccRCC^34^. While the syngeneic RENCA tumour model^35^ has been used in hundreds of published studies to investigate different aspects of renal cell carcinoma biology, including tumour cell-immune cell interactions and responses to checkpoint therapies, the extent to which this model reflects human ccRCC remains unclear as tumours derived from these cells do not histologically resemble ccRCC tumours nor do they harbour the characteristic genetic mutations of human ccRCC.

To begin to address this problem of lack of appropriate mouse models, in this study we showed that the VpR mutant autochthonous mouse ccRCC model is resistant to anti-PD-1 plus anti-CTLA-4 checkpoint therapy, alone or in combination with other agents. While this study was in preparation, another publication described a new mouse ccRCC model based on CRISPR-Cas9 mediated combinatorial mutation of *Vhl*, *Pbrm1*, *Keap1* and *Tsc1* that is also similarly resistant to single, double and triple anti-PD-1, anti-CTLA-4 and HIF-2α inhibition therapies^36^. It is therefore noteworthy that two independent immunocompetent autochthonous mouse models that are based on two different sets of mutations are resistant to immune checkpoint therapy and to HIF-2α inhibitors, which both have therapeutic effects in some but not all human ccRCCs. Potential reasons for the absence of therapeutic effects in the mouse models could relate to the rapid growth of ccRCCs in the mouse models compared to the slower growth of human ccRCC, there could simply be fundamental differences between mice and humans in the biology of ccRCC, or that the mouse models might oversimplify the genetic complexity of the human disease. Speaking against these arguments are the many molecular and cellular overlaps between the mouse models and human ccRCC^19,20,36^ as well as the fact that the VpR model and the *Vhl/Pbrm1/Keap1/Tsc1* model are therapeutically sensitive to mTOR inhibition^19,36^, which induces therapeutic effects in at least some cases of human ccRCC and the VpR model is also sensitive to anti-VEGF sunitinib therapy^19^, which is also efficacious in some human ccRCCs. These studies provide evidence that the mouse models reproduce at least some aspects of the therapeutic sensitivity of human ccRCCs. We therefore believe that these models might potentially inform about mechanisms underlying specific resistance to immune checkpoint therapies. Several lines of evidence outlined below lead us to speculate that PD-1- and/or CTLA-4-based immune checkpoint therapies may in fact not be the optimal way to manipulate anti-tumour immunity in the context of the specific immune microenvironmental and tumour cell features of ccRCC.

One hint is our observation that despite the absence of tumour shrinkage and the fact that checkpoint therapy even reduced the number of T cells in the tumour microenvironment, we identified that splenic CD4^+^ and CD8^+^ T cells from immune checkpoint inhibitor treated animals, but not from untreated animals, could recognise ccRCC cells isolated from the same animal in a co-culture setting. This ex vivo syngeneic mixing experiment reflects a systemic effect of the therapy that would otherwise have been hidden, and implies that the checkpoint therapy does induce some degree of anti-tumour T cell activity. The fact that T cell activation was only apparent once the T cells and ccRCC cells were removed from the tumour microenvironment implicates the in vivo environment as a suppressor of T cell activation in response to checkpoint blockade. Single cell RNA-seq of the tumour microenvironment suggested that multiple myeloid lineage-derived cell types and cell states may contribute to T cell suppression. Importantly, there are many similarities between the mouse ccRCC model and human ccRCC tumours in terms of infiltration by myeloid-lineage cells. scRNA-seq revealed that VpR ccRCC tumours are infiltrated by a population of cells that express many genes that are characteristic of PMN-MDSC cells which could contribute to suppression of T cells, including *Arg2*, *Ptgs2*, *Tgfb1*, *Il1b* and *Cd300ld*. The proteins encoded by these genes are all pharmacologically targetable. Consistent with the scRNA-seq, our flow cytometry and immunohistochemical analyses confirmed that a Ly6G^+^ putative PMN-MDSC population of cells is present in VpR ccRCCs. Several studies have identified neutrophil-like cells in human ccRCCs and showed that high intratumoural neutrophil infiltration correlates with adverse outcome^37,38^, or that high intratumoural or systemic neutrophil to lymphocyte ratios correlate with poor responses to immunotherapy^39,40^. Our scRNA-seq, flow cytometric and immunohistochemical analyses also revealed strong inflammation by cells of the monocyte-macrophage lineage. In scRNA-seq we identified a cluster of monocyte-derived cells with features of mis-differentiation towards an M-MDSC-like, TAM2-like phenotype. These cells show low expression of genes involved in antigen presentation and elevated *Ly6c2* expression, consistent with an immature MDSC/macrophage phenotype, expression of the immunosuppressive gene *Tgfb1* as well as of genes that were previously shown to characterise TAM2-like macrophage populations in human ccRCC^13–16^, including *Trem2*, *C1qa*, *Apoe*, *Fn1*, *F13a1* and *Gas7*.

Another important conclusion from our data is that ligands of T cell immune checkpoint receptors other than PD-1 and CTLA-4 are highly abundantly expressed by ccRCC cells and myeloid cells. scRNA-seq and bulk RNA-seq datasets revealed that there are strong molecular similarities between mouse and human ccRCC in terms of the expression patterns of immune checkpoint ligands. Analysis of published ccRCC proteomic datasets also support this idea. Genes encoding the ligands of PD-1 are very weakly expressed by tumour cells and myeloid cells, consistent with the absence of a strong response to anti-PD-1 therapy. The most abundant checkpoint ligand in terms of both absolute expression levels as well as being expressed by tumour cells and by different types of infiltrating myeloid cells is *Lgals3/LGALS3*, encoding GALECTIN-3, a ligand for the LAG-3 immune checkpoint receptor. Numerous clinical trials involving inhibition of LAG-3 in combination with other immune modulatory agents are currently ongoing^28^. Specific inhibitors of GALECTIN-3 have also been developed and these may represent an attractive alternative therapeutic strategy to block LAG-3 signalling as well as other potentially pro-tumourigenic biological functions of GALECTIN-3 such as promoting angiogenesis, tumour cell intra- and extravasation and metastasis^41^. A clinical trial of the small molecule GALECTIN-3 inhibitor GB1211 combined with the PD-L1 inhibitor atezolizumab is currently ongoing for non-small cell lung carcinoma (NCT05009680). Two different TIM-3 ligands are also strongly and widely expressed in mouse and human ccRCC. *Hmgb1/HMGB1* is expressed by ccRCC cells and myeloid cells in mouse and human tumours and *Lgals9/LGALS9*, encoding GALECTIN-9 is expressed by myeloid cells in both mouse and human ccRCC. Several ongoing clinical studies for ccRCC are testing TIM-3 inhibitors combined with PD-1 inhibitors^28^. HMGB1 is released by damaged cells and acts as a potent pro-inflammatory signal through a number of receptors including RAGE, TLR family receptors, CXCR4, IL-1R1, as well as TIM-3^42^. Blockade of extracellular HMGB1 using several different inhibitors in multiple orthotopic syngeneic mouse models of basal-like breast cancer and non-small cell lung cancer inhibited tumour growth, altered the tumour microenvironment to a more favourable anti-tumour state and showed enhanced therapeutic efficacy in combination with PD-1 inhibition^43^. PVR is a ligand of the TIGIT immune checkpoint receptor and is upregulated at the mRNA level in the mouse ccRCC model as well as at the protein level in human ccRCC, suggesting that blocking TIGIT activation could also be investigated for ccRCC therapy. A clinical trial testing anti-TIGIT + anti-PD-L1 + belzutifan for ccRCC is ongoing (NCT04626479). Finally, CD276 (B7-H3) is another member of the B7 family of immune checkpoint ligands which functions as an inhibitor of T cell activation, although the receptor for this ligand has not yet been identified^44^. Comparing normal kidney to ccRCC we show that CD276 expression is upregulated in mouse and human tumours as shown by bulk RNA-seq as well as through proteomic analyses in human ccRCC. Early clinical trials of agents that block CD276 gave been initiated in small cell lung carcinoma and in other solid tumours^45^. A recent study implicated aberrant mTOR activation as a factor that activates CD276 expression and showed that neutralising antibodies against CD276 decreased renal tumour growth in an autochthonous mouse model driven by *Tsc2* mutation^46^, providing strong evidence that CD276 might also play a role in ccRCC, a tumour type which is known to be characterised by high levels of mTOR activation^47^. Collectively, these observations suggest that the abundant and widespread expression of several different immune checkpoint systems beyond PD-1 and CTLA-4 may act to suppress T cell activation in the context of the ccRCC microenvironment.

Our characterisation of the *Vhl/Trp53/Rb1* mutant mouse ccRCC model provides an experimental system that will allow the systematic dissection of the effects of individual and combined inhibition of these different checkpoint ligand/checkpoint receptor signalling systems on T cell activation in ccRCC. A major advantage of studying autochthonous mouse models over human ex vivo systems such as kidney cancer organoid or fragmentoid cultures is that ccRCC tumours co-evolve with the immune system over a period of 6 to 18 months and reproduce all of the relevant systemic features of the anti-tumour immune response including interactions of immune cells in lymph nodes and the spleen, as well as potential barriers to immune infiltration such as blood vessels and extracellular matrix of the tumour. A practical limitation of these mouse studies is that they are very time-consuming, requiring regular imaging of each animal to identify the onset of tumours on a mouse-by-mouse basis. This limits the scale of the analyses and number of different therapeutic combinations that can be feasibly tested. Since the *Vhl/Trp53/Rb1* mutant mouse ccRCC model does not generate metastases, it also does not allow investigation of the effects of immune checkpoint inhibitor therapy on metastatic disease, which may have a different immune microenvironment to the primary tumour. These limitations notwithstanding, the many similarities that we have identified between this model and human ccRCC suggests that these pre-clinical studies are worthwhile to try to speed up efforts to identify and prioritise therapies that could then be tested in the even longer timeframe of human clinical studies. A final potential limitation of our analyses is that even though we have previously shown that there are strong similarities between the VpR ccRCC model and human ccRCC at the transcriptomic and proteomic levels^19,20^, and now show that there are also large overlaps in the cellular and molecular features of the tumour immune microenvironment and expression of immune checkpoint ligands, we cannot exclude that the genetics of the mouse model may not be reflective of all human ccRCCs. While *VHL* is mutated in the vast majority of human ccRCCs and *TP53* is mutated in 15% of ccRCC metastases^48^, *RB1* is almost never mutated^49,50^. The *Vhl/Trp53/Rb1* mutant genotype rather reflects the combinations of chromosomal copy number gains and losses that affect cell cycle regulatory genes in about two-thirds of human ccRCC^19^. We believe that some of the immunological features of this model will be reflected in all or most ccRCCs but also that there will likely be other features that are unique to specific mutational combinations and potentially to specific sub-regions of ccRCC tumours^16^. Nonetheless, our current study argues that it would be worthwhile in future research efforts to carry out similar analyses in other mouse models of ccRCC^29–33^ that are driven by different combinations of driver mutations to attempt to identify genotype-dependent contributions to the tumour immune microenvironment and to responses to different immune modulatory therapies. Obtaining this type of detailed understanding of the fundamental rules that govern immunological responses in ccRCC tumours, combined with the availability of defined mouse tumour models that allow the experimental manipulation of the underlying mechanisms, will allow a more rational approach to designing the next generation of immune modulatory therapies that are better tailored to the features of ccRCC in general and potentially more specifically to each patient’s tumour.

## Materials and methods

### Mouse tumour studies

Renal epithelial cell-specific deletion of *Vhl*, *Trp53* and *Rb1* was induced in a mouse strain that was established by our laboratory^19^. These mice were generated by intercrossing *Ksp1.3-CreER*^*T2*^ (B6.Cg background)^51^, *Vhl*^*fl/fl*^ (C;129S background)^52^, *Trp53*^*fl/fl*^ (FVB;129P2 background)^53^ and *Rb1*^*fl/fl*^ (FVB;129P2 background)^54^ mice. Male and female *Ksp1.3-CreER*^*T2*^*; Vhl*^*fl/fl*^*; Trp53*^*fl/fl*^*; Rb1*^*fl/fl*^ mice were fed tamoxifen-containing food (400 mg/kg tamoxifen citrate) for two weeks beginning at 6 weeks of age as previously described^19^. Ultrasound imaging (sonography) every 3–4 weeks starting 5 months post-feeding was conducted as described^55^ to detect tumour onset in each animal. At that timepoint, mice were imaged on a weekly basis using magnetic resonance imaging (MRI) to calculate accurate tumour volumes as described^55^. Therapy was initiated once the largest tumour in an animal reached a volume of between 5 and 20 mm^3^. Smaller tumours that were present in the animal were also followed by MRI throughout the duration of the therapy. Mice were sacrificed once the largest tumour reached a volume of 200—1000 mm^3^ or after a maximum of 14 weeks of therapy. When material permitted, tumour samples were digested for flow cytometry analyses and were fixed for histological analyses. Tumour-bearing mice were randomized and injected intraperitoneally three times a week with 10 mg/kg with α-PD-1 (clone: RMP1-14, Bio X Cell (BE0146)) plus α-CTLA-4 (clone: 9H10, Bio X Cell (BE0131)), or with rat IgG2a isotype control (clone: 2A3, Bio X Cell (BE0089)) plus polyclonal Syrian hamster IgG (Bio X Cell (BE0087)). For combination therapies with α-PD-1 plus α-CTLA-4 mice were also treated with either PT-2385 (MedChemExpress (HY-12867), 20 mg/kg oral gavage, 1x/day, 5x/week), Acriflavine (Sigma (A8126, 2 mg/kg intraperitoneal injection, 2x/day, 5x/week), α-GR-1 (Bio X Cell (BE0075), 10 mg/kg intraperitoneal injection, 3x/week), Cabozantinib (Selleckchem (S1119), 30 mg/kg oral gavage, 1x/day, 5x/week) or Cisplatin (Selleckchem (HY-17394), 2.5 mg/kg intraperitoneal, 1x/week). All experimental protocols were approved under the license G17/112 of the Regierungspräsidium Freiburg and all methods were carried out in accordance to these regulations. Animal experiments are reported according to the ARRIVE guidelines.

### ccRCC cell culture

VpR ccRCC cell lines generated in this study were made as previously described^55^.

### Splenocyte freezing and T cell culture

The spleen was removed and placed on a 100 μm filter in a 10 cm-Petri dish with MACS buffer. The spleen was squashed with a syringe stamp through the filter, followed by a second 100 μm filter step into a 50 mL Falcon tube. Splenocytes were centrifuged at 1,200 rpm for 10 min and the pellet re-suspended in MACS buffer and added to a 96-well plate for flow cytometry analyses. For freezing of the splenocytes, they were centrifuged (1200 rpm for 8 min) and frozen in 90% FCS with 10% DMSO. For thawing, the splenocytes were slowly thawed under warm constant water flow and put into a 10 cm-Petri dish with RPMI medium + murine IL-2 (50 IU/mL, 12340024, Immuno Tools). On the following day, the splenocytes were either stained and/or subjected to T cell activation. Splenocytes cells were stained with Cell Trace Violet Proliferation Dye. Cells were centrifuged at 1200 rpm for 8 min in a 50 mL Falcon tube and the pellet was re-suspended in 1 × PBS (1 × 10^6^ cells/mL) containing 5 mM Cell Trace Violet, incubated at 37 °C in the dark for 20 min. Afterwards, the 50 mL Falcon tube was filled with RPMI + 10% FCS to inactivate the staining dye and left at room temperature protected from light for 5 min. For T cell activation, 25 mL of anti-CD3/anti-CD28 Dynabeads per 1 × 10^6^ cells were placed in a 2 mL Eppendorf tube and washed by addition of 1 mL of PBS and vortexing. Dynabeads were then collected on one side of the tube by placing it in a magnetic stand, allowing the removal of the wash solution. Further, the anti-CD3/anti-CD28 Dynabeads were re-suspended in the same volume of proliferation medium (RPMI + 10% FCS) as the initial volume of magnetic beads taken (25 mL of anti-CD3/anti-CD28 Dynabeads per 1 × 10^6^ cells). The splenocytes were then re-suspended in the proliferation medium with the magnetic beads and murine IL-2 (50 IU/mL) was added to the tube. Cells were then added in the co-culture studies to mouse VpR ccRCC cells and assayed after 3 days by flow cytometry.

### Tissue and cell preparations for flow cytometry

Tumour biopsies or biopsies of tumour-free kidney were cut into small pieces in 5 mL HBSS, pipetted up and down vigorously, transferred to a 50 mL tissue culture flask with washing with 40 mL HBSS. 5 mL 10 × Triple Enzyme Mix (100 mg Hyaluronidase from bovine testes, Sigma-Aldrich, H3506; 1 g Collagenase from Clostridium histolyticum, Sigma Aldrich, C5138; Deoxyribonuclease I from bovine pancreas, Sigma-Aldrich, D5025; in 100 mL HBSS) were added to the 45 mL of tumour suspension and this was shaken at 80 rpm at room temperature for 1 h. In order to better dissociate the tumour cells the suspension was pipetted up and down every 10–15 min. After 1 h incubation the cell suspension was centrifuged at 50 g at RT for 10 min to remove debris, the supernatant collected and the pellet discarded. The supernatant was centrifuged at 200 g for 5 min and the pellet was washed with 10 mL wash buffer (1 g BSA and 2 mL 0.5 M EDTA with 800 mL HBSS) at 200 g for 5 min. To remove red blood cells, the solution was incubated with 1 mL of ACK lysing buffer for 1 min, filled up with 10 mL HBSS and centrifuged again at 200 g for 5 min. Spleens were dissected and put on a 100 μm filter in a Petri dish with MACS buffer. Spleens were then pushed through the the filter with the help of a syringe stamp and the filtrate in the Petri dish was taken and filtered again through a 100 μm filter into a 50 ml Falcon tube. This was centrifuged at 1,200 rpm for 10 min and afterwards the pellet was re-suspended. Dissected tibias and femurs were separated at the knee joint and the bones were cleaned from the remaining flesh with a scalpel. The two tibiae and two femurs were put in a Petri dish filled with 1× PBS. If additional bone marrow was needed, the same procedure was performed with the forelegs. To flush the bone marrow cavity, one bone was taken and cut on both sides in order to access the area of the red bone marrow. 1× PBS was sucked into a syringe with a needle and it was pushed slowly into the bone marrow cavity. This was flushed gently with PBS until the cavity turned whitish. After collecting the bone marrow cells in the Petri dish, they were filtered through a 100 μm filter into a 50 mL Falcon tube. The remaining parts on the filter were pushed through using a syringe stamp. The Falcon tube was centrifuged at 1,200 rpm for 8 min and the supernatant was removed. The pellet was re-suspended in fresh 1× PBS or MACS buffer. Peripheral blood was centrifuged in a 1.5 mL Eppendorf tube and the supernatant was mixed with 500 μL-1 mL ACK lysing buffer, depending on the amount of blood, and incubated for 1 min. Afterwards, this was centrifuged again and the colour of the pellet was checked. If the pellet was white with a little bit of red colour the blood samples were resuspended for flow cytometry. If the pellet was still red, a red blood digestion with ACK lysing buffer was repeated again.

### Flow cytometry

Samples from the different tissues were divided into three different wells, two for the three different staining panels (Panels 1 and 2, see below) and one for the unstained control. Where necessary, tissue samples were also prepared for Fluorescence Minus One (FMO) controls in separate wells. As a first step the 96-well plate was centrifuged at 1600 rpm for 5 min at 4 °C and the wells were re-suspended in 50 μL LIVE/DEAD^™^ Fixable Aqua Dead Cell Stain (pre-diluted 1:500 in 1 × PBS) in order to stain dead cells by incubation for 20 min at 4 °C and in the dark. Afterwards, 120 μL MACS buffer was added to each well to wash away the stain and the 96-well plate was centrifuged at 1600 rpm for 5 min at 4 °C. Subsequently, the pellets in the wells were re-suspended in 25 μL Fc Block (pre-diluted 1:25 in MACS buffer) and incubated for 10 min at 4 °C, in the dark. After 10 min, 25 μL antibody mix was added additionally to the Fc Block solution and the cells incubated for 30 min at 4 °C and in the dark. The cells were twice washed with MACS buffer and the intracellular staining proceeded in specific wells. Following the extracellular staining, intracellular staining was conducted by re-suspending the pellets in 200 μL of FoxP3 Fixation/Permeabilization Buffer and incubating the cells in this solution for 30–60 min at 4 °C. Afterwards, the cells were spun down and washed twice in 200 μL 1× Permeabilization Buffer. Further, the cell pellets were re-suspended in 100 μL 1× Permeabilization Buffer and 1 μL of FoxP3 antibody added to each well. The cells were incubated in this antibody solution for at least 30 min at room temperature. Finally the cells were spun down again and washed two times with 200 μL 1 × Permeabilization Buffer. After these two final washing steps, the pellets were re-suspended in MACS buffer and the cells measured using a flow cytometer.

#### Panel 1

Mouse α-CD45.2-APCeFluor780 (104, eBiosciences, 1:100 dilution), rat α-CD3-Pacific Blue (17A2, BioLegend, 1:200 dilution), mouse-α-CD4-PerCP-Cy5.5 (OKT4, BioLegend, 1:400 dilution), rat α-CD8a-FITC (53-6.7, BD Biosciences, 1:200 dilution), rat α-FoxP3-PE (FJK-16 s, eBioscience, 1:200), rat α-PD-1-PE-Cy7 (RMP1-30, eBioscience, 1:400 dilution), rat α-B220-AlexaFluor647 (RA3-6B2, BioLegend, 1:800 dilution) rat α-CD68-BV605 (FA-11, BioLegend, 1:400 dilution).

#### Panel 2

Mouse α-CD45.2-APCeFluor780 (104, eBiosciences, 1:100 dilution), rat α-F4/80-AlexaFluor488 (BM8, BioLegend, 1:200 dilution), rat α-CD11b-Pacific Blue (M1/70, BioLegend, 1:400 dilution), rat α-Ly-6G-PE-Cy7 (1A8, BioLegend, 1:800 dilution), rat α-Ly-6C-PerCP-Cy5.5 (AL- 21 (RUO), BD Biosciences, 1:200 dilution), rat α-CD115-PE (AFS98, BioLegend, 1:800 dilution), rat α-PD-L1-APC (10F.9G2, BioLegend, 1:200 dilution) rat α-CD68-BV605 (FA-11, BioLegend, 1:400 dilution).

#### Panel 3

Mouse α-CD45.2-APCeFluor780 (104, eBiosciences, 1:100 dilution), mouse-α-CD4-PerCP-Cy5.5 (OKT4, BioLegend, 1:400 dilution), rat α-CD8a-APC (53-6.7, BioLegend, 1:200 dilution), rat α-Granzyme B-PE-Cy7 (NGZB, eBioscience, 1:100 dilution), rat α-IFN-γ-AlexaFluor488 (XMG1.2, BioLegend, 1:100 dilution).

#### Panel 4

α-CD45.2-APCeFluor780 (104, eBiosciences, 1:100 dilution), mouse-α-CD4-PerCP-Cy5.5 (OKT4, BioLegend, 1:400 dilution), rat α-CD8a-FITC (53-6.7, BD Biosciences, 1:200 dilution), rat α-PD-1-PE-Cy7.

### Immunohistochemistry

Immunohistochemical stainings were conducted using antibodies and protocols described in^20^.

### Single cell RNA-sequencing

To prepare samples for single cell RNA-Seq (scRNA-seq) the tumour was dissected and digested, stained with LIVE/DEAD^™^ Fixable Aqua Dead Cell Stain and with mouse α-CD45.2-APCeFluor780 (104, eBiosciences, 1:100 dilution) as described above. Cell sorting was performed using a BD Aria Fusion with a nozzle size of 70 μm. The sorting was performed for living CD45^+^ and CD45^-^ cells. After sorting, the cells were washed twice by centrifugation at 300 rcf for 5 min. The pellet was re-suspended in 1 ml 1 × PBS with 0.04% BSA by using a wide-bore pipette tip and gently pipetting up and down. The cells were transferred into a 1.5 mL Eppendorf to avoid any further loss and were centrifuged at 300 rcf for 5 min again. The supernatant was removed, the pellet re-suspended again and the tube with the cells centrifuged down. In the final step of this protocol, the pellet was re-suspended in 1 mL 1 × PBS with 0.04% BSA and the cells were manually counted in a Neubauer chamber then mixed in the ratio of 3 CD45^+^ cells to 1 CD45^-^ cell to generate a suspension of 1000–1100 cells/μL. Depending on the targeted cell recovery a specific volume of the cell stock concentration was mixed with a specific volume of nuclease-free water. In the case of 10,000 cells 15–16.5 μl of cell stock were mixed with 28.2 – 26.7 μl of nuclease-free water, respectively. The protocol of the Chromium Next GEM Single Cell 3ʹ Reagent Kits v3.1 protocol (10× Genomics) was followed to generate single cell libraries and the samples sent for sequencing.

### Bioinformatic analyses of single cell RNA-sequencing

Processing of barcodes, alignment to the reference genome, and single cell gene counting was performed using the Cell Ranger software (version 4.0.0) from 10× Genomics (10× Genomics, Pleasanton, CA, USA). Downstream analyses were performed using Seurat (v4)^56^ within the R framework. Only cells with a least 200 features and a mitochondrial content below 25% were considered. This initial high mitochondrial content cutoff was chosen due to the fact that proximal tubule epithelial cells have a naturally high mitochondrial content. Differential gene expression analyses were used in a second step to identify clusters of leukocytes with relatively high mitochondrial content (low quality cells) that were then excluded from further analyses. Features expressed in less than three cells were excluded from the analysis. The data were log-normalized with a scale factor of 10,000 taking the mitochondrial content into account as regression parameter during normalization and scaling with the sctransform^57^ workflow. Dimensionality reduction was done using principal component analysis (PCA) with 50 considered dimensions, followed by a batch correction with the harmony algorithm^58^ taking the sample origin into account. The neighborhood graph was assessed using the first 50 batch corrected principal components. Unsupervised clustering was performed using the Louvain algorithm. Differentially expressed features for each cluster were calculated as implemented by the MAST algorithm^59^. Clusters were labeled according to specific markers identified in the DE analysis. Heatmaps are plotted with scaled normalized expression values per feature (row). The heatmpas were created with R (v4.3.2) and the package pheatmap (v1.0.12). This analysis, as well as the complete dataset, are available online in a web browser-based, publicly accessible searchable database and visualisation package called scExplorer (https://nephgen-intern.imbi.uni-freiburg.de). scExplorer allows querying of the expression of single genes or of lists of genes in all clusters or in selected clusters, in pooled or separated samples. Data can be visualised and downloaded in the form of violin plots, number and percentage of cells expressing the gene, cluster average gene expression heatmaps or UMAP-projected gene expression heatmap plots. Raw scRNA-seq data have been uploaded to GEO with the identifier GSE259361. Processed scRNA-seq data from 8 human ccRCC samples were downloaded to scExplorer from the study of Bi et al.^15^.

### Bioinformatic analyses of human ccRCC RNA-seq and proteomics

Analyses of bulk human RNA-seq of the TGCA KIRC dataset and of proteomics of ccRCC samples from the Clinical Proteomic Tumor Analysis Consortium (CPTAC) were conducted using the Ualcan platform^60^. Data used in this publication were generated by the National Cancer Institute Clinical Proteomic Tumor Analysis Consortium (CPTAC).

## Supplementary Information

Supplementary Information.

## Data Availability

Raw scRNA- seq data have been uploaded to GEO with the identifier GSE259361.
