# Supplementary material for: Characterisation of an autochthonous mouse ccRCC model of immune checkpoint inhibitor therapy resistance
Source: Sci Rep. 2025 Jun 5;15:19818. doi: 10.1038/s41598-025-04917-1 (PMC12141660; doi:10.1038/s41598-025-04917-1)
Supplement: Supplementary file 1 — Supplementary Information. [file 41598_2025_4917_MOESM1_ESM.pdf]

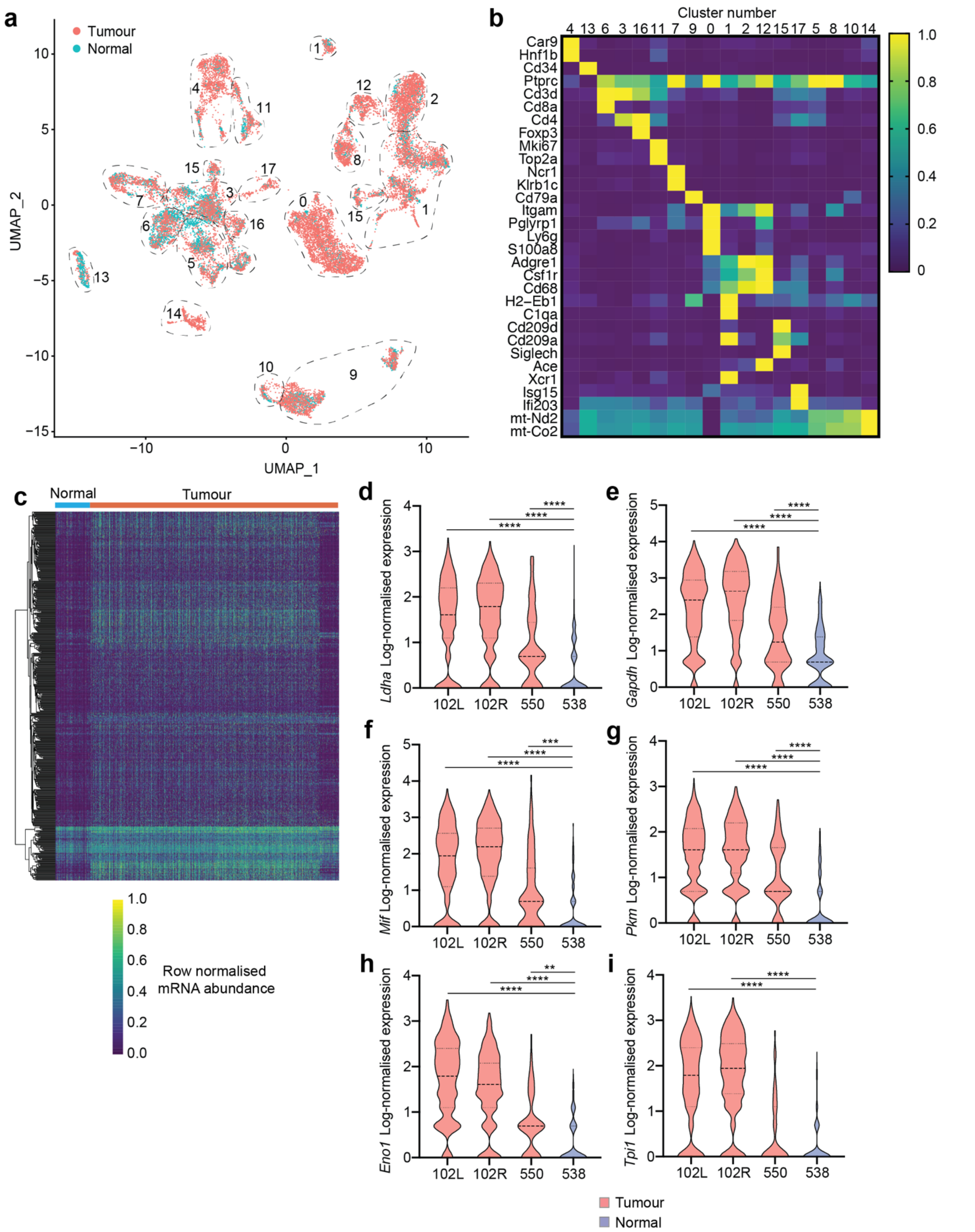

Supplementary Fig. 1: a. UMAP plot depicting cellular distributions from one normal kidney and three ccRCC tumours. **b.** Gene expression heatmap of selected genes that are specific to individual cellular lineages. **c.** Heatmap of differentially expressed genes between normal and tumour cells from cluster 4. **d-i.** Violin plots of the expression of selected HIF-α target genes in cluster 4 (epithelial cells and ccRCC cells) from scRNA-seq of mouse normal kidney and VpR ccRCC. *P* values were calculated using Kruskal-Wallace non-parametric one-way ANOVA with Dunn's multiple comparisons test.

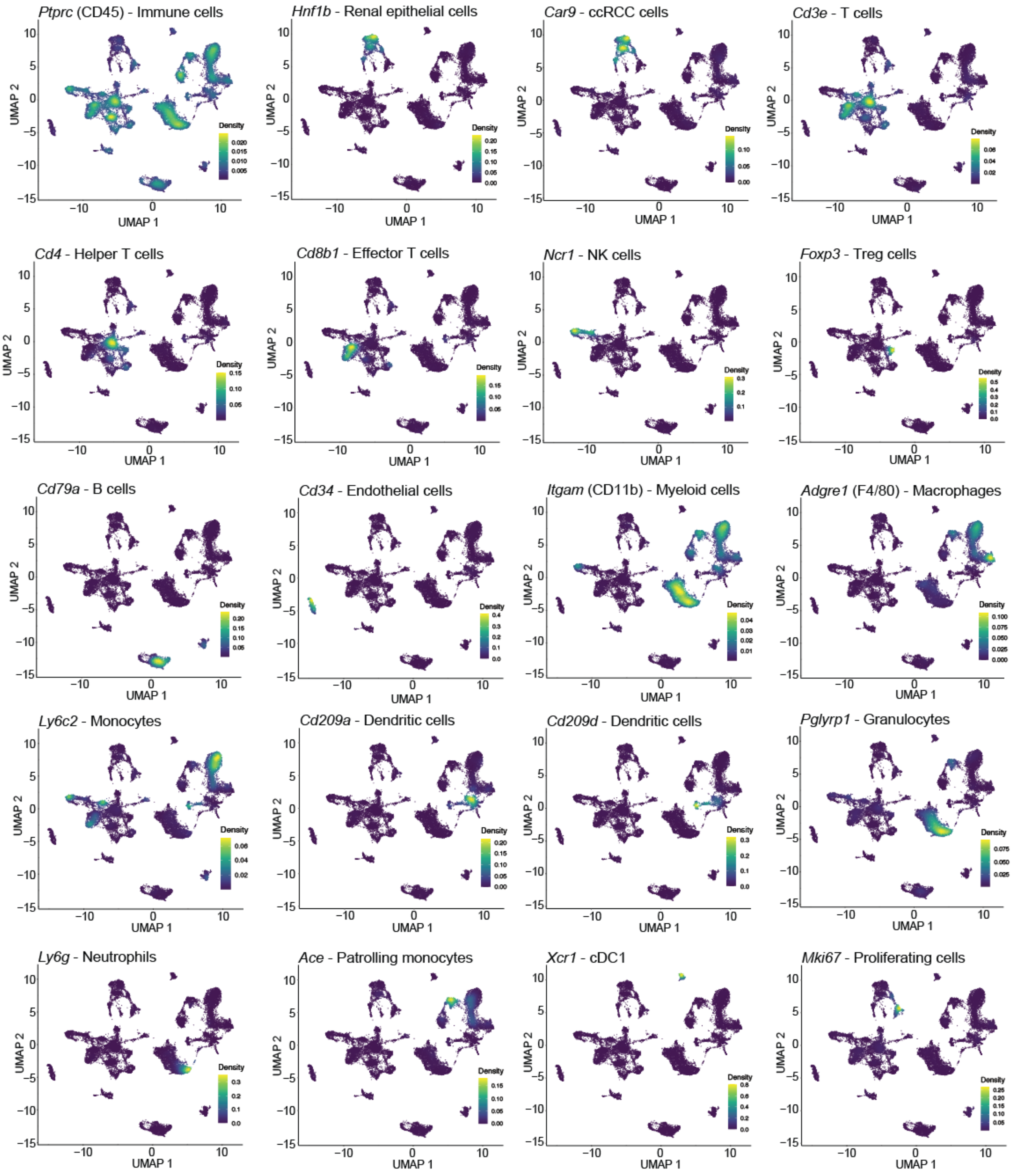

Supplementary Fig. 2: Expression of indicated genes that are specific to the indicated cell types in UMAP plots of scRNA-seq of normal kidney and VpR ccRCC tumours.

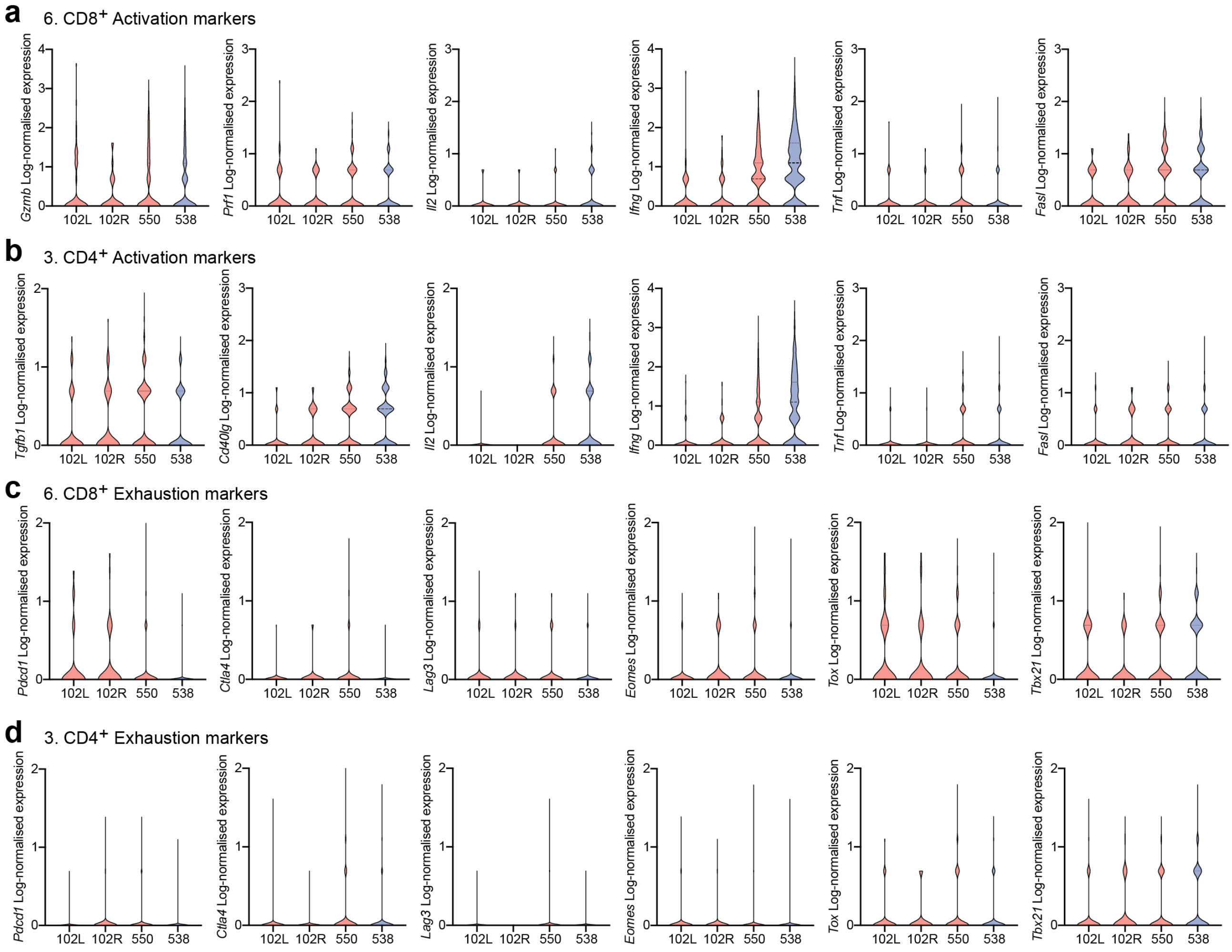

Supplementary Fig. 3: a-d. Violin plots of the expression of selected genes that reflect $CD8^+$ T cell activation (**a.**), $CD4^+$ T cell activation (**b.**) or T cell exhaustion (**c,d.**) in the $CD8^+$ T cell cluster 6 (**a,c.**) or the $CD4^+$ T cell cluster 3 (**b,d.**) from scRNA-seq of mouse normal kidney and VpR ccRCC. Kruskal-Wallace non-parametric one-way ANOVA revealed that there were no statistically significant differences in gene expression between normal and tumour samples.

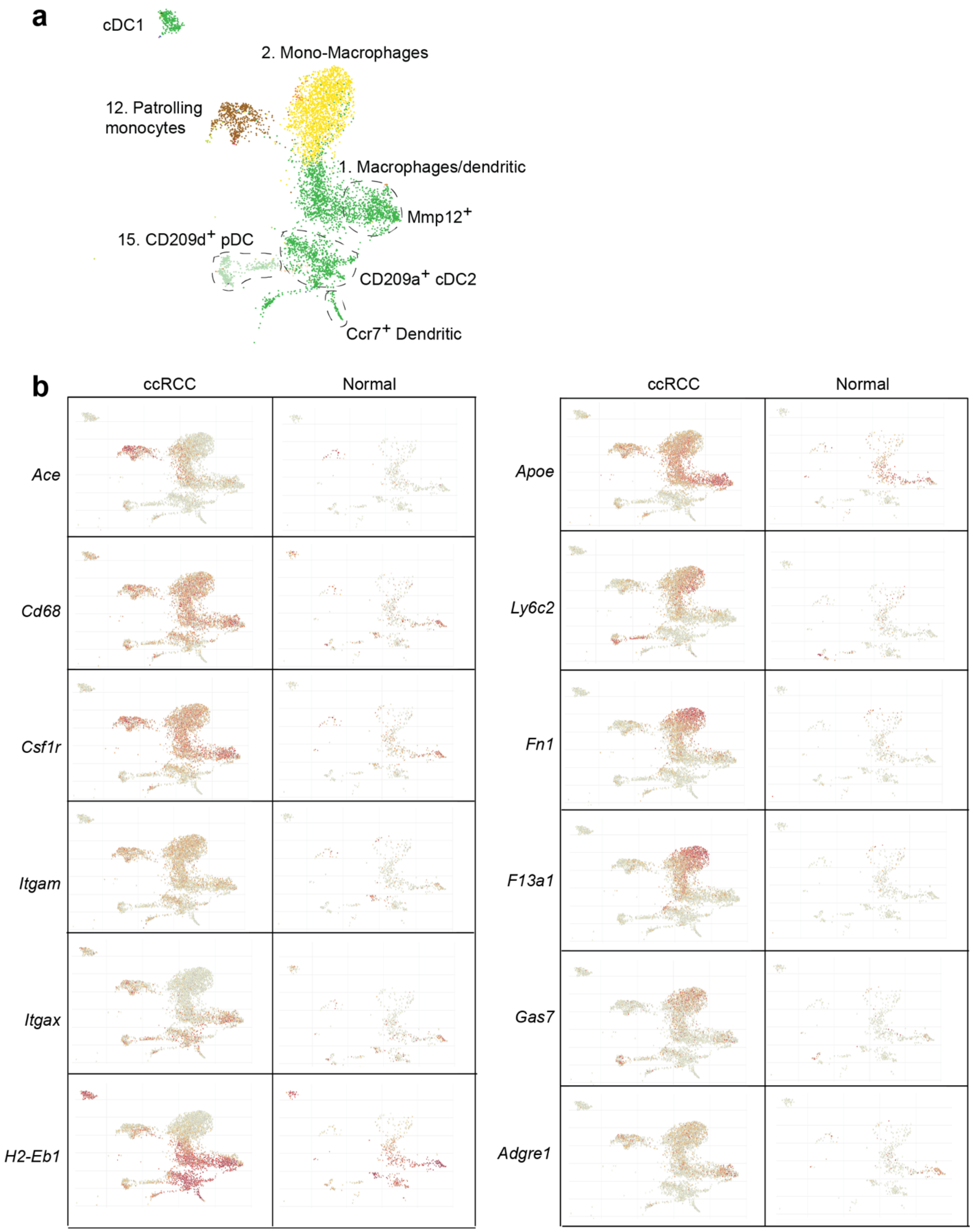

Supplementary Fig. 4: a. UMAP plot of monocyte, macrophage and dendritic cell cluster. **b.** Expression of indicated genes projected onto UMAP plots of scRNA-seq of normal kidney and VpR ccRCC tumours. Red colour indicates increased gene expression.
